# Rare gastroesophageal junction tumors or cysts of bronchial origin: A case report

**DOI:** 10.1097/MD.0000000000042216

**Published:** 2025-05-30

**Authors:** Yunfei Zhao, Yuan Ma, Ling Yang, Renmei Wu, Linwei Zhao, Hongyan Jiang

**Affiliations:** a Department of Pathology, Suining Central Hospital, Sichuan, China; b Department of Thoracic Surgery, Yunnan Cancer Hospital, Kunming, Yunnan, China; c Department of Ultrasound, Suining Central Hospital, Sichuan, China; d Department of Radiological Imaging, Suining Central Hospital, Sichuan, China.

**Keywords:** bronchogenic cyst, gastro-esophageal junction, gastrointestinal mesenchymal tumor

## Abstract

**Rationale:**

Bronchogenic cysts (BCs) are rare congenital anomalies that often present without distinct clinical symptoms, complicating their diagnosis. However, as these cysts enlarge, they can cause symptoms due to compression of adjacent tissues and organs. In February 2024, a 32-year-old male was admitted to the Central Hospital of Suining City, Sichuan Province, China, with a preliminary diagnosis of a “gastrointestinal mesenchymal tumor.” A thorough pathological evaluation ultimately confirmed the diagnosis as a BC. The cyst was successfully excised through surgical intervention.

**Patient concerns:**

The patient reported experiencing discomfort in the left upper abdomen for over a year.

**Diagnosis:**

On January 2, 2024, surgery was performed under general anesthesia. Intraoperative exploration revealed no metastatic tumors in the pelvis, mesentery, or liver. A tumor measuring approximately 5 cm by 6 cm was identified on the lesser curvature of the cardia, with firm consistency and clear resection margins. No perigastric lymph node enlargement was observed. A proximal gastric resection was performed, and postoperative pathological analysis confirmed the diagnosis of a BC.

**Interventions:**

Postoperative care included anti-infective therapy, nutritional support, and acid-suppressive rehydration therapy.

**Outcomes:**

The patient, a young male with no prior medical conditions, recovered well without wound infections and was discharged successfully. Follow-up assessments have shown sustained positive health outcomes.

**Lesson:**

BCs are rare, particularly at the gastroesophageal junction, and are often misdiagnosed as gastrointestinal mesenchymal tumors. Accurate differentiation is crucial, and surgical excision remains the most effective treatment.

## 1. Introduction

Bronchogenic cyst (BC) is a rare congenital abnormality, rooted in the intricate deviations of early embryogenesis. This anomaly is hallmarked by a cystic structure, consequent of the malformation within the primitive foregut, lending it an unique distinction in the spectrum of developmental disorders.^[1]^ BCs are commonly identified incidentally during physical examinations or consultations for unrelated conditions, often presenting without specific clinical manifestations. Utilizing a comprehensive array of diagnostic modalities such as computed tomography (CT), magnetic resonance imaging, and ultrasound can facilitate precise localization of the cyst and its adjacent structures. The size and anatomical location of BCs play a pivotal role in determining the associated symptomatology. Enlarged cysts have the potential to exert pressure on neighboring organs and tissues, resulting in symptoms such as discomfort, nausea, vomiting, and other manifestations. In cases of cyst infection, patients may additionally present with fever and significant pain.^[2]^ However, the majority of BCs are considered benign lesions. Cysts may also develop in the pericardium, esophagus, and other regions as a result of aberrant germ cell shedding and migration. As reported, stomach cysts originating from the bronchi are rare, and BCs at the gastro-esophageal junction are especially infrequent.^[3]^ A case of BC at the gastro-esophageal junction was diagnosed in the Department of Pathology of Suining Central Hospital on January 3, 2024. A rare case report was performed after departmental consultation and in order to enhance learning and communication.

## 2. Case presentation

### 2.1. Patient information

A 32-year-old male patient was admitted to the hospital for 1 year with persistent left upper abdominal discomfort and subsequently diagnosed with hepatic-gastric occupancy. He described there was no family history and occasional smoking and alcohol consumption. The patient is young and currently works freelance without exposure to occupational-related diseases. It recorded the pain intensified from February 2022 and radiating to the back. Notably, he did not show other symptoms like nausea, vomiting, bloating, or diarrhea.

### 2.2. Diagnostic imaging, blood tests, histopathological assessment, etc

#### 2.2.1. CT scan

CT scan, a round soft tissue density lesion near the gastric cardia at the small gastric curvature in the liver and gastric fissure was found, which was suspected as a gastrointestinal stromal tumor. Two weeks later, the patient’s condition worsened, and he went to the hospital for abdominal CT examination, which showed a mass on the small bend of the cardia (compared with previous), and gastrointestinal stromal tumor was highly suspected. Referred to the General Surgery Department of Suining Central Hospital, the patient was in normal mental condition, with no swollen superficial lymph nodes, flat and soft abdomen, no abdominal tenderness, rebound pain, and no liver enlargement. Or the spleen is located below the chest cavity, negative for Murphy sign, and no percussion pain in the liver or kidney area.

#### 2.2.2. Blood tests

Routine blood tests were unremarkable; tumor marker tests showed: alpha fetoprotein 1.69 (‐), carcinoembryonic antigen (‐), CA19-9 (‐), CA72-4 (+), total prostate-specific antigen (‐), free prostate-specific antigen (‐), fP/tP% (‐). Mycoplasma pneumoniae IgM antibody test was negative; general bacterial smears, antacid bacilli smears, and fungal smears examined did not find any bacteria; the no fungi were found, and no antacids were found.

#### 2.2.3. Ultrasound

**Figure 1.**
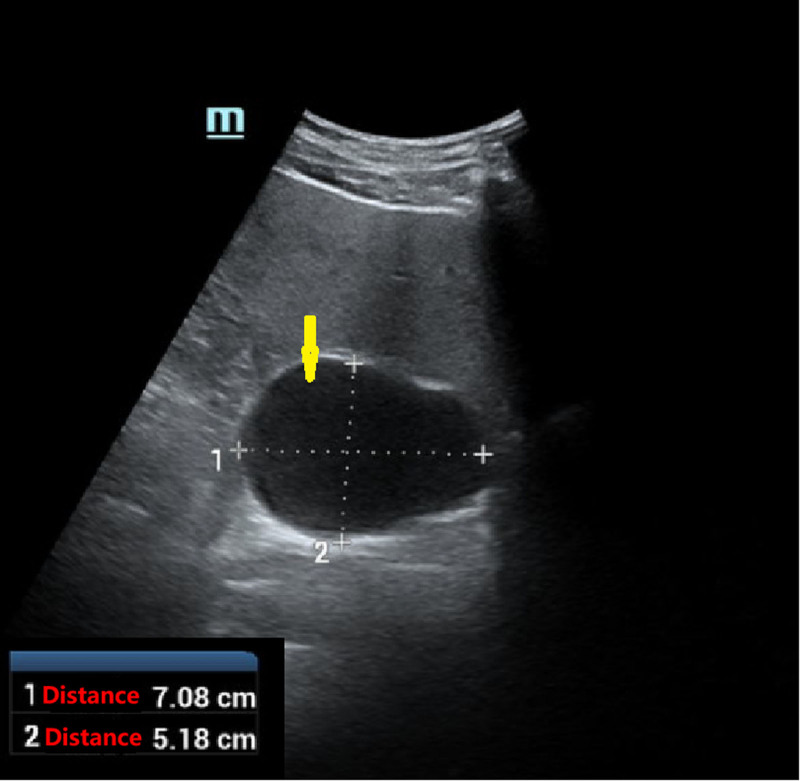
Ultrasound showed a cystic occupancy detected adjacent to the caudate lobe of the liver, suspected to be of hepatic origin, with a cyst measuring approximately 7.1 cm × 5.2 cm. the yellow arrow points to the cyst.

A cystic occupancy was detected adjacent to the caudate lobe of the liver, measuring approximately 7.1 cm × 5.2 cm, with clear borders and regular morphology; CDFI: no significant blood flow signal was seen within it, and cystic occupancy adjacent to the caudate lobe of the liver: it was necessary to exclude whether it was of hepatic origin (Fig. 1).

#### 2.2.4. Enhanced CT

**Figure 2.**
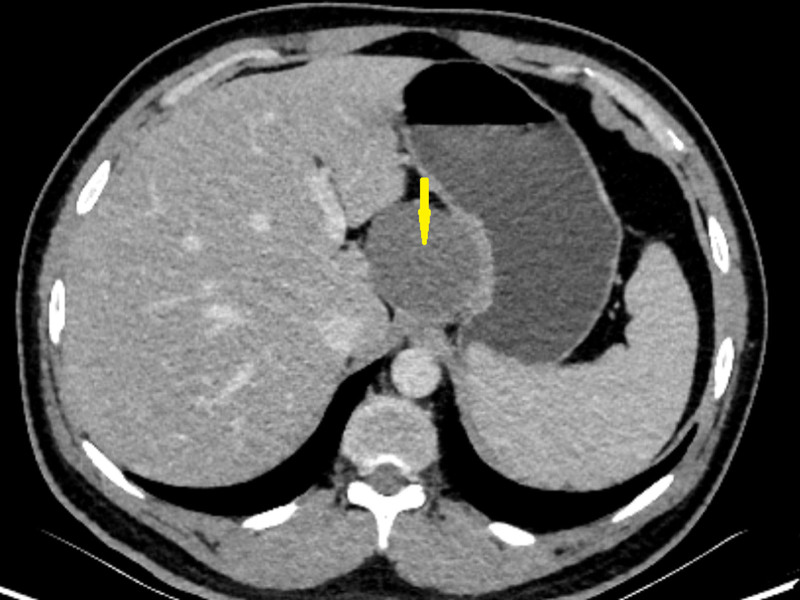
Enhanced CT. A mass-like slightly low-density shadow in the hepatogastric hiatus, about 5.8cm × 4.7cm in size, mild homogeneous enhancement of the lesion on enhanced scan, suspected mesenchymal stromal tumor. The yellow arrow points to this tumor. CT = computed tomography.

A mass-like slightly low-density shadow was seen in the hepatogastric hiatus, measuring about 5.8 cm × 4.7 cm, with clear and smooth borders of the lesion, adjacent to the lesser curvature of the stomach; the enhancement scan of the lesion was mildly and more homogeneously strengthened, and gastrointestinal mesenchymal tumor was considered, which needed to be ruled out (Fig. 2).

The supervising surgeon communicated with the patient preoperatively, who was more emotionally stable, and informed the patient of the possible intraoperative risks, and the patient expressed his understanding and readiness to proceed with the surgery. Then, after a thorough consultation with the patient and their family, a decision was reached to surgically excise the mass. Following this, a series of preoperative examinations were diligently conducted. On January 2, 2024, the surgical intervention was carried out under general anesthesia. During the surgical procedure, it was meticulously observed that the tumor was located along the lesser curvature of the cardia, with partial attachment to the lower esophagus. The tumor measured approximately 7 cm by 6 cm in size and exhibited a firm consistency, regular shape, and clearly defined margins. Additionally, no enlargement was noted in the perigastric lymph nodes. Consequently, a proximal gastric major resection was successfully performed.

#### 2.2.5. Macroscopic examination

**Figure 3.**
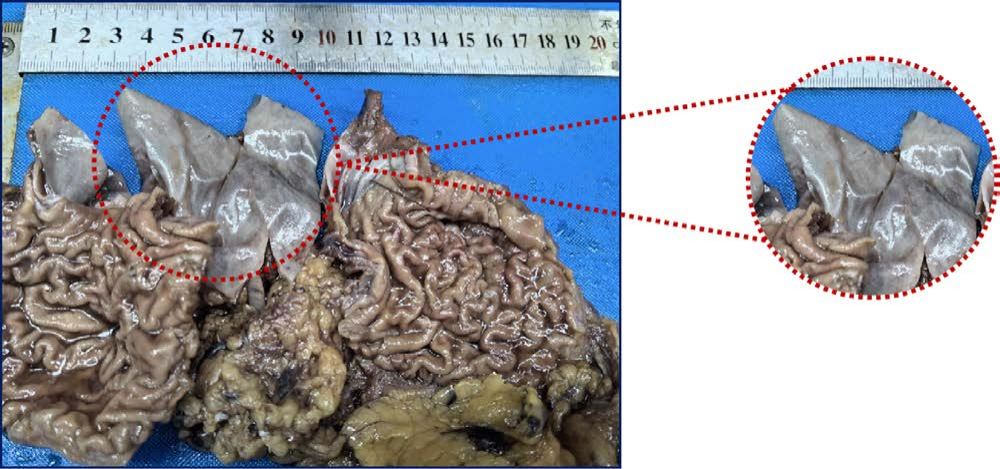
Macroscopic examination. proximal stomach of the greater omentum and esophagus, with a cystic mass visible submucosally at the gastro-esophageal junction within a red dotted circle.

The presented anatomy includes a proximal stomach, accompanied by a segment of the greater omentum and a portion of the esophagus, without any dissection. The stomach measured 12 cm in length, 9 cm in width, and 6 cm in depth. The distinction between the greater and lesser curvatures of the stomach was indistinct. The esophagus had a length of 0.8 cm, situated immediately superior to the upper incision margin and 3 cm away from the lower incision margin. At the gastroesophageal junction, a cystic mass was observed with dimensions of 7 cm in length, 5 cm in width, and 4.5 cm in depth. The cross-section of the mass revealed a unilocular cystic structure, featuring a wall thickness of 0.1 cm to 0.2 cm. The inner wall of the cyst was smooth and contained a gray-brown, sticky, jelly-like substance, clearly distinct from the surrounding tissue (Fig. 3). The mucosa of both the stomach and esophagus in the area of the mass appeared smooth. The perigastric region and the greater omentum showed no evidence of enlarged nodules.

#### 2.2.6. Microscopic observation

**Figure 4.**
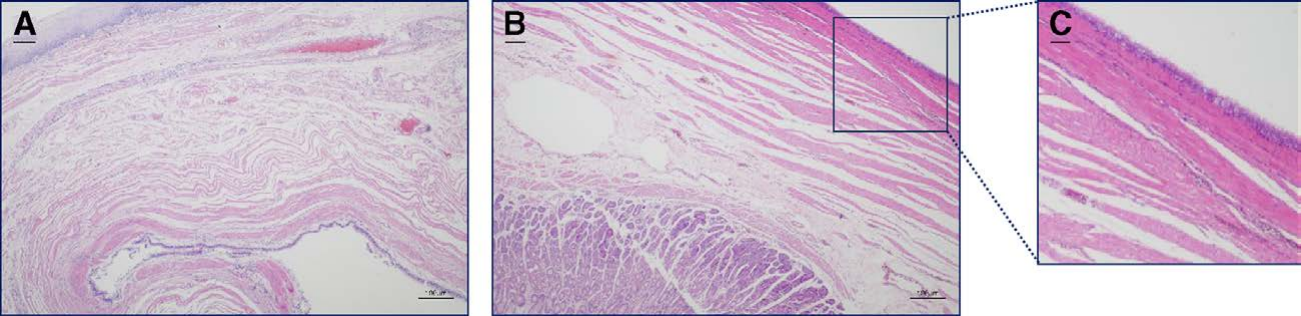
Hematoxylin eosin staining. Microscopically, the tumor was located in the lamina propria of the stomach and esophagus, the section was cystic and the cyst wall was covered with ciliated columnar cells, cup cells and basal cells. (A) The tumor was partially located in the lamina propria of the esophagus. (B) The tumor was partially located in the lamina propria of the stomach wall. (B) The tumor is partially located in the lamina propria of the gastric wall. (C) The cyst wall is covered with ciliated columnar cells, cup cells and basal cells. (C) is a partial enlargement of (B). Scale bar = 100 µm.

Tissue treatments involved in morphological observations in this study were referred to our previous study.^[4]^ Microscopically, the tumor was situated within the muscularis propria at the point where the stomach and esophagus meet. The section displayed a cystic nature, with the capsule wall being covered by ciliated columnar cells, cup cells, and basal cells. A portion of the tumor extended into the muscularis propria of the esophagus (Fig. 4A), while another part infiltrated the muscularis propria of the gastric wall (Fig. 4B). Zooming in on a portion of Fig. 4B is visible, as Fig. 4C revealed that the cystic wall was indeed lined by these ciliated columnar cells, cup cells, and basal cells.

#### 2.2.7. Immunohistochemistry

**Figure 5.**
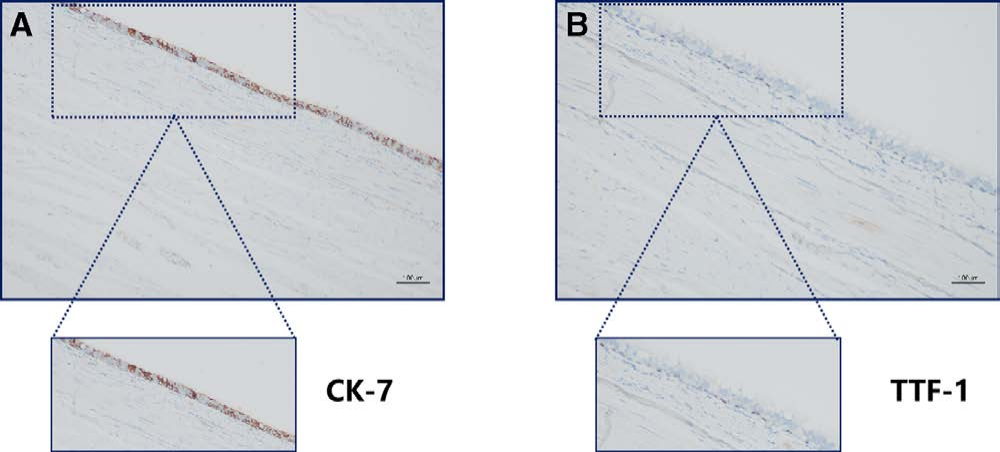
Immunohistochemical staining for markers Cytokeratin20, Cytokeratin 7, Napsin A, Thyroid Transcription Factor-1, Villin. As shown in this figure. positive immunohistochemical results, where (A) is Cytokeratin 7, (B) is Thyroid Transcription Factor-1. Scale bar = 100 µm.

Immunohistochemical staining was performed on representative sections of 3 µm in thickness, and immunohistochemistry was performed on a Roche BenchMark ULTRA IHC machine (Roche Diagnostics). Immunohistochemical staining was performed using the following antibodies at 37 °C for 60 minutes: Cytokeratin 20 (cat. no. EP23), Cytokeratin 7 (cat. no. EP16), Napsin A (cat. no. IP64), thyroid transcription factor-1 (cat. no. SPT24), Villin (cat. no. EP163). The primary antibodies were all ready‑to‑use antibodies from OriGene Technologies, Inc., Rockville, USA, and were added manually during the run without dilution. The secondary antibody (UV HRP UNIV MULT; 8 minutes at 37 °C) was detected using the UltraView Universal DAB Detection Kit system [(10)K01773; Roche Tissue Diagnostics, Oro Valley, USA]. DAB was used as the color developer (8 minutes at 37 °C), hematoxylin (37 °C; 8 minutes) was used for re‑staining after color development and Roche Bluing reagent was used for re‑bluing. The films were sealed with neutral gum and then read and imaged under an Olympus light microscope. Immunohistochemical results showed (Fig. 5): Thyroid Transcription Factor-1 (basal cells +), Cytokeratin 7 (ciliated columnar cells +), Napsin A (‐), Cytokeratin20 (‐), Villin (‐) (negative results were not demonstrated in the figure).

### 2.3. Diagnostic and follow-up

#### 2.3.1. Pathological diagnosis

BC of the “gastro-esophageal junction.”

#### 2.3.2. Follow‑up

After the operation, the patient returned to the ward successfully, and was treated with anti-infection, nutritional support, acid-suppressing rehydration, and symptomatic treatment after the operation. Patients should be prohibited from eating a small amount of liquid diet for the first 3 days after surgery and a moderate amount of liquid diet for 7 to 10 days after surgery. Patients are advised to get out of bed for moderate activities for about 3 days after surgery. Given that the patient was a young male with no underlying disease, the patient recovered well and had no wound infection. The patient had an uneventful postoperative period and was successfully discharged from the hospital, and the patient is doing well on follow-up to date.

## 3. Discussion

The respiratory system initially originates from the primitive foregut, which elongates the primitive trachea towards the ventral mesoderm in the 4th week of the embryo and gradually branches out to form the tracheobronchial tree,^[5]^ and in the process of differentiation, the histiocytes of the primitive trachea, which have been detached and migrated to other parts of the body, may be born as ectopic bronchiolar cysts at the corresponding sites. Ectopic bronchial cysts are rare, and bronchial cysts appear to grow on the stomach wall to form cysts of gastro-bronchial origin, a rare lesion first reported in 1956.^[6]^ Gastro-bronchogenic cysts usually lack specific laboratory findings and imaging manifestations, and the clinical misdiagnosis rate of this disease is as high as 40% to 60%,^[7]^ which needs to be confirmed by pathologic examination. Gastric bronchogenic cysts usually lack specific laboratory results and imaging findings, and are often detected on physical examination or imaging studies for other reasons. The diagnosis of gastro-bronchogenic cysts may be difficult to confirm by patient history, signs, laboratory and even imaging studies. In recent years, a number of studies have found a possible correlation between elevated levels of the tumor markers glycan chain antigen, such as, CA-125, CA72-4, CA19-9 and gastro-bronchogenic cysts.^[8–10]^ In this study, the patient was accompanied by an elevated CA72-4 level, a normal CA19-9 level, and no CA-125 examination was performed. Therefore, when encountering the above symptoms in the subsequent diagnosis, the examination of CA-125, CA72-4, and CA19-9 should be perfected, and if there is an elevation of the above indexes, the gastro-bronchogenic cyst needs to be alerted. In this study, the patient was finally diagnosed with a BC at the gastro-esophageal junction despite the elevation of one indicator, which further demonstrates that clinical symptoms, blood tumor markers and pathological diagnosis need to be combined in clinical diagnosis to ensure the correctness of the diagnosis.

The patient in this study was initially suspected of having a gastrointestinal mesenchymal tumor prior to surgery. However, intraoperatively, the mass was found to lack the characteristic features of a gastrointestinal mesenchymal tumor, such as brittleness and propensity for hemorrhage. Instead, the mass exhibited cystic characteristics, leading to its classification as a cyst rather than a gastrointestinal mesenchymal tumor. Subsequently, a complete excision was performed on the cyst to minimize the risk of recurrence.^[11]^ The literature suggests that surgical resection is the most efficacious treatment for bronchial cysts, with early intervention being particularly advantageous for patients experiencing symptoms related to tumor compression. During surgery, it is imperative for the surgeon to exercise caution in order to prevent damage to the cyst wall and to achieve complete resection, as incomplete removal may heighten the risk of recurrence.^[12]^ Therefore, postoperative follow-up is usually required to determine whether it is a recurrence of the tumor based on the patient’s symptoms and imaging manifestations.

Bronchial cysts develop during embryonic development due to the migration and shedding of tissue cells, typically remaining in close proximity to their original location. Consequently, abdominal cysts originating from the bronchi are uncommon, particularly those situated within the gastric wall.^[13]^ Dewing et al first described very rare bronchial cysts close to the gastro-esophageal junction area in 1956.^[6]^ Patients with cysts of gastro-bronchial origin have an age of onset of 25 to 8l years, the incidence in women is about twice as high as in men, and they usually occur in the fundus of the stomach or cardia.^[1,14]^ In contrast, the patient in this report was a young male with a cyst occurring at the gastro-esophageal junction, which is much rarer and has a better scientific and literature reporting value.^[15]^ Gastric bronchogenic cysts have potential for malignant transformation.^[16–18]^ Resection is recommended once the lesion is detected, and surgical removal will improve symptoms of cyst compression and reduce the risk of BCs turning into malignant tumors.^[16]^ However, whether surgical intervention is warranted in asymptomatic patients remains controversial, but the patient’s specific situation should be considered, and conservative management may be preferable in patients of advanced age with underlying disease.

It has been suggested that chronic inflammation of the gastric mucosa caused by BCs may lead to gastric adenocarcinoma.^[19]^ We therefore need to improve the diagnosis of BCs, with early diagnosis and prompt treatment, but accurate preoperative diagnosis is challenging, and most patients are easily misdiagnosed as gastrointestinal mesenchymal tumor. Therefore, this study firstly discusses the differential diagnosis between gastro-esophageal bronchogenic cysts and gastrointestinal mesenchymal tumors. Gastric mesenchymal tumors are prevalent in middle-aged and elderly people, and may be located in the submucosa, subplasma membrane, or the intermuscular layer of the gastric wall. The lesions are solid, and enhancement scans are helpful in differentiating them, but it is difficult to differentiate them when the tumor is completely cystic. As BCs are rare and clinicians lack knowledge of this disease, which is one of the reasons for the failure to obtain an accurate preoperative diagnosis of this disease, we need to report this case in order to strengthen the communication and exchange with the industry. In addition, BCs in the gastro-esophageal junction should be differentiated from the following diseases: (1) smooth muscle tumor: the tumor is mostly solid, with few cystic changes and calcifications. (2) Gastric ectopic pancreas: the cystic nature of ectopic pancreas can be manifested due to its different pathological composition, but the swollen lesion is widely connected with the gastric wall, and the characteristic umbilical concavity sign can be seen, and the lesion is located in the gastric antrum and the gastric body.

In conclusion, the scarcity of BCs and the unique nature of the condition have resulted in limited understanding. This study focuses on BCs in the gastro-esophageal junction area, with the goal of advancing knowledge and expanding the literature on this condition to facilitate interdisciplinary dialogue.

### 3.1. Study limitations

This study has several limitations. First, as a single-case report, the findings lack generalizability and cannot represent broader clinical patterns. Second, preoperative imaging (CT and ultrasound) initially misdiagnosed the cyst as a gastrointestinal stromal tumor, reflecting challenges in radiologic differentiation of rare cystic anomalies. Third, tumor marker assessment was incomplete (e.g., CA-125 was not evaluated), potentially limiting diagnostic correlations. Fourth, follow-up duration and long-term outcomes (e.g., recurrence risk) remain unspecified. Additionally, the patient’s demographic profile (young male without comorbidities) differs from typical BC populations (older adults and females), which may restrict applicability. Finally, the study did not explore conservative management options, a critical consideration in asymptomatic cases. Future multicenter studies with extended follow-up and advanced molecular analyses could address these gaps.

## Author contributions

**Conceptualization:** Yunfei Zhao.

**Data curation:** Yuan Ma.

**Methodology:** Linwei Zhao, Renmei Wu.

**Project administration:** Ling Yang.

**Supervision:** Yunfei Zhao.

**Writing – original draft:** Hongyan Jiang, Yunfei Zhao.

**Writing – review & editing:** Hongyan Jiang.
